# CT-based deep learning prediction of complete response in intermediate-stage hepatocellular carcinoma treated with drug-eluting beads transarterial chemoembolization

**DOI:** 10.1093/bjrai/ubaf018

**Published:** 2025-11-21

**Authors:** Jérémy Dana, Armine Vardazaryan, Benoit Gallix, Maxime Ronot, Jean-Paul Mazellier, Marlee Parsons, Valérie Vilgrain, Thomas F Baumert, Caroline Reinhold, Nicolas Padoy, Jules Grégory

**Affiliations:** Département de Radiologie, Radio-oncologie et Médecine Nucléaire, Université de Montréal, Montréal, Québec, H3T 1J4, Canada; Imagerie et Ingénierie, Centre de Recherche du Centre Hospitalier de l'Université de Montréal, Montréal, Québec, H2X 0A9, Canada; Département de Radiologie, Centre Hospitalier de l'Université de Montréal, Montréal, Québec, H2X 0C1, Canada; Université de Strasbourg, Institut Hospitalo-Universitaire (IHU) Strasbourg, Strasbourg, 67000, France; Department of Diagnostic Radiology, McGill University, Montreal, H4A 3J1, Canada; Département de Radiologie, Hôpital Américain de Paris, Neuilly-sur-Seine, 92000, France; Inria, Institut National de Recherche en Sciences et Technologies du Numérique, Paris, 75013, France; Department of Radiology, FHU MOSAIC2, APHP Hôpital Beaujon, CRI Inserm 1149, Université Paris Cité, Clichy, 92110, France; Université de Strasbourg, Institut Hospitalo-Universitaire (IHU) Strasbourg, Strasbourg, 67000, France; Department of Diagnostic Radiology, McGill University, Montreal, H4A 3J1, Canada; Department of Radiology, FHU MOSAIC2, APHP Hôpital Beaujon, CRI Inserm 1149, Université Paris Cité, Clichy, 92110, France; Inserm U1110, Institut de Recherche sur les Maladies Virales et Hépatiques, Université de Strasbourg, Strasbourg, 67000, France; Pôle Hépato-digestif, Service d’Hépatogastroentérologie, Hôpitaux Universitaires de Strasbourg, Strasbourg, 67000, France; Department of Diagnostic Radiology, McGill University, Montreal, H4A 3J1, Canada; Research Institute of the McGill University Health Centre, Augmented Intelligence & Precision Health Laboratory (AIPHL), Montreal, H4A 3J1, Canada; Université de Strasbourg, Institut Hospitalo-Universitaire (IHU) Strasbourg, Strasbourg, 67000, France; Department of Radiology, FHU MOSAIC2, APHP Hôpital Beaujon, CRI Inserm 1149, Université Paris Cité, Clichy, 92110, France

**Keywords:** transarterial chemoembolization, hepatocellular carcinoma, treatment response, deep learning, liver imaging score

## Abstract

**Objectives:**

To develop a CT-based deep learning (DL) model to predict complete response (CR) to drug-eluting beads-transarterial chemoembolization (TACE) in patients with naive Barcelona Clinic Liver Cancer (BCLC) B hepatocellular carcinoma (HCC).

**Methods:**

This dual-centre retrospective study included 93 patients with BCLC B HCC treated with drug-eluting beads-TACE (median size of 40 mm and 37 mm at Institutions 1 and 2). Complete response was defined as per modified Response Evaluation Criteria in Solid Tumours on liver contrast-enhanced CT within 2 months of treatment. A twin-network DL model with spatio-temporal Video Vision Transformer (ViViT) architecture was developed to predict CR using baseline dedicated liver CT. The model was extensively trained/tested based on an 8-fold cross-validation approach with an ensemble technique, a model vote system where the outcome is the average of multiple model predictions.

**Results:**

The CR rate was 36% (18/50) and 22% (11/49) at Institutions 1 and 2. The model showed high specificity and AUC, as well as moderate sensitivity and balanced accuracy when using either the late arterial phase (0.91 ± 0.12, 0.86 ± 0.16, 0.43 ± 0.23, and 0.67 ± 0.13, respectively) or the portal venous phase (0.90 ± 0.15, 0.85 ± 0.17, 0.57 ± 0.30, and 0.74 ± 0.16, respectively).

**Conclusion:**

The developed CT-based DL model predicted CR to drug-eluting beads-TACE in patients with naive BCLC B HCC with high specificity. It should be refined to improve sensitivity.

**Advances in knowledge:**

The study underscores the potential of artificial intelligence in precision medicine in patients with HCC. While the model shows promise, further research with larger datasets and prospective studies is needed to enhance its predictive power and clinical applicability.

## Introduction

The current guidelines from the European Association for the Study of the Liver endorse TransArterial Chemoembolization (TACE) as the primary treatment for managing intermediate-stage hepatocellular carcinoma (HCC) patients, characterized by multinodular tumours, preserved liver function, and satisfactory performance status.^1^ Despite these criteria, this intermediate stage encompasses a diverse patient presentation, displaying varying tumour burdens and residual liver functionality. The heterogeneity manifests in inconsistent response rates to TACE, with median overall survival durations fluctuating between 13 and 43 months.^2^^,^^3^

The updated Barcelona Clinic Liver Cancer (BCLC) criteria from 2022 have narrowed the application of TACE to a more specific patient subgroup with well-defined tumour nodules, preserved portal flow, and selective vascular access, excluding those eligible for liver transplantation under the expanded Milan criteria and those with diffuse disease.^4^ This refined patient selection process underlines the need for more nuanced prognostic tools to predict therapeutic outcomes. Such tools would streamline the identification process of patients poised to derive maximum benefit from TACE, thereby optimizing the clinical outcome-to-cost ratio.^5^ Complete response (CR) to TACE is a recognized prognostic indicator associated with improved overall prognosis,^6^^,^^7^ yet the ability to predict which patients will achieve CR remains elusive. Indeed, several treatment alternatives, including adjuvant immunotherapy, could be considered in the absence of predicted CR on pre-therapeutic imaging.

The diverse patient responses within the intermediate stage of HCC suggest that conventional linear prediction models may not be sufficient.^8–10^ These models often overlook the complex genetic and phenotypic variations of HCC.^11^ Only a few imaging studies have attempted to go beyond simple quantitative and qualitative tumoural assessment, including tumour size and number, the presence of arteries in the tumour or the tumour’s apparent heterogeneity. A more comprehensive image-driven analysis could provide insights into the likelihood of CR to TACE, enabling personalized treatment strategies.

Artificial Intelligence (AI), specifically deep learning (DL), has emerged as a powerful tool for the intricate analysis required for such predictions. Prior research has demonstrated the feasibility of employing DL algorithms to anticipate HCC responses to TACE. Still, these have been limited by the inclusion of advanced-stage HCC patients in the datasets,^12^ potentially skewing the results.

This study aims to develop a contrast-enhanced CT-based DL model to predict CR to DEB-TACE in patients with naïve HCC categorized in the second subgroup BCLC stage B.

## Methods

This dual-centre retrospective study was approved by the respective Research Ethics Boards of the 2 university hospitals (REB 2023-9568 & CERIM). Given the study’s retrospective nature, the informed consent requirement was waived.

### Patient selection

**Figure 1.**
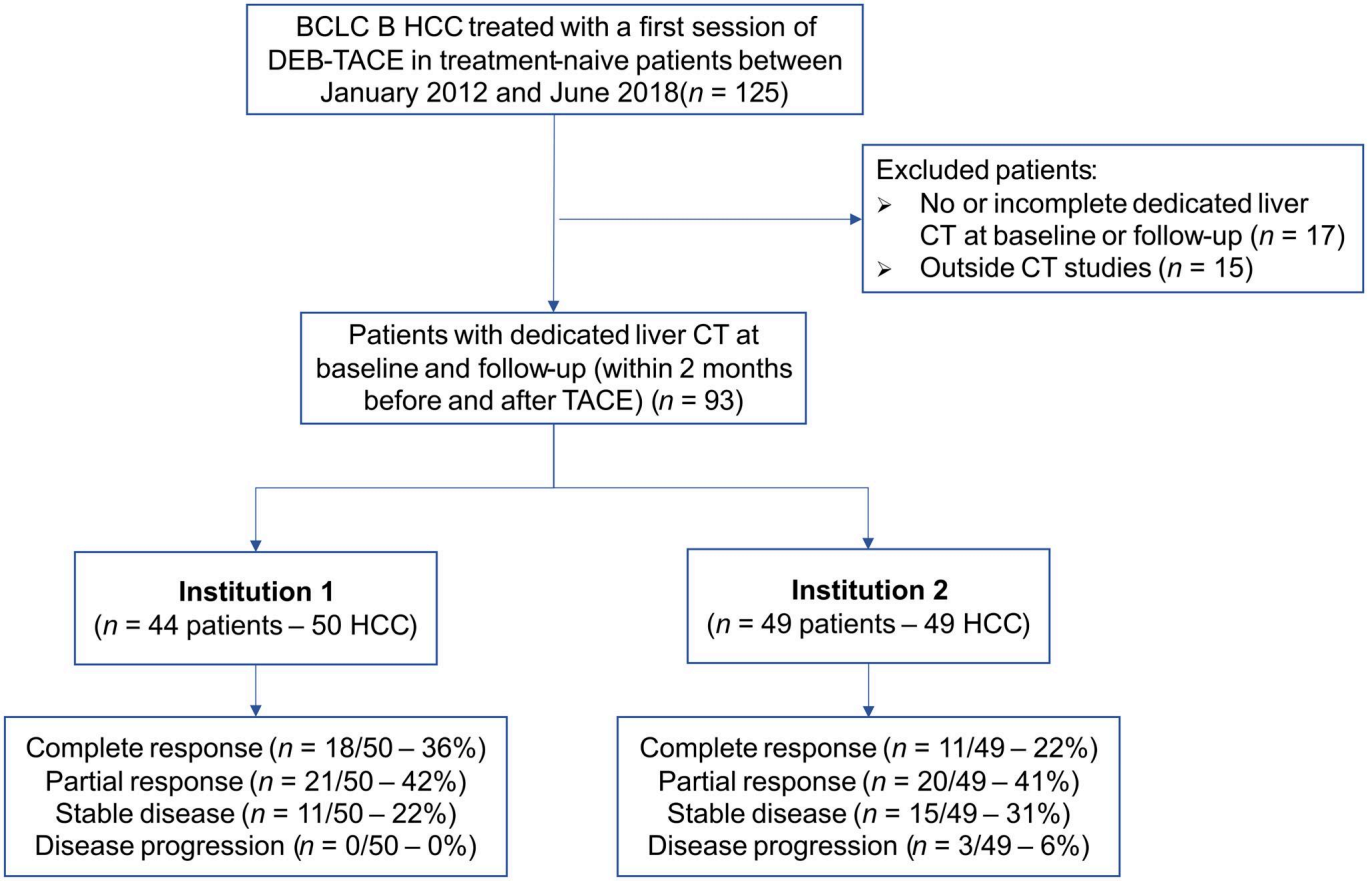
Flowchart. BCLC = Barcelona clinic liver cancer; HCC, hepatocellular carcinoma; DEB-TACE = drug-eluting bead transarterial chemoembolization.

The study cohort consisted of consecutive patients with BCLC stage B intermediate-stage HCC [Well-defined nodules, preserved portal flow, selective access], diagnosed by histopathology or as per LI-RADS criteria^13^ (LI-RADS 5), who underwent a first DEB-TACE treatment between January 2012 and June 2018 (Figure 1). Patients were included in this study if they were aged 18 years or older, had received a first session of DEB-TACE, without any previous or concurrent HCC treatment, and had undergone both baseline and follow-up contrast-enhanced CT scans within a specified 2-month window around their treatment dates.

### Imaging protocol

All patients underwent baseline and follow-up CTs according to the institutions’ protocol, consisting of multiphase contrast-enhanced CT scans, including late arterial, portal venous and delayed phases, scheduled within 2 months before and following the TACE, respectively. Given the retrospective and dual-centre nature of the study, as well as the long inclusion period, CT scans were obtained on a variety of 64 multiple detector CTs (GE Healthcare, Chicago, Illinois, USA; Philips, Andover, MA, United States) and several contrast material were used (iopromide, 300 mg/mL, Ultravist 300, Bayer Schering Pharma AG, Berlin, Germany; iohexol, 300 mg iodine/mL, Omnipaque 300, GE Healthcare, Chicago, IL, United States; iomeprol, 350 mg iodine/mL, Iomeron 350, Bracco Imaging, Milano, Italy). All protocol requirements for contrast-enhanced CT imaging met the criteria recommended by the AASLD guidelines.^14^ Specific technical criteria were required for inclusion in the study: (1) multiphasic CT including late arterial phase (35-40 seconds after contrast material administration or, if bolus tracking software had been used, 20 seconds after the attenuation in the abdominal aorta reached the predefined threshold of 100 HU), PV phase (35 seconds after the late arterial phase) and delayed phase (5 minutes after contrast material administration); (2) injection into an antecubital vein of iodinated contrast material adapted to patient’s weight (cm³/kg with a maximum of 120 cm³) at flow rate ranging from 2 to 3 cm³/seconds; (3) image thickness and image interval of 1.25 mm.

### TACE procedure

After a multidisciplinary tumour board identified DEB-TACE as an appropriate treatment for each patient, segmental or subsegmental DEB-TACE was performed by interventional radiologists with more than 10 years of experience. Prior to embolization, angiography of the hepatic and mesenteric artery was performed to map the liver vascular anatomy, check for arteriovenous shunts, and identify arterial feeders of the tumour (s). Segmental arteries were catheterized selectively in large lesions, while for smaller lesions—if feasible—the catheterized vessels were generally subsegmental branches. In segmental or subsegmental embolization, a microcatheter was always used (2.7 Fr; Progreat; Terumo, Europe N.V, Leuven, Belgium) to avoid spasm and allow better-controlled delivery of the suspension with the blood flow. A microcatheter was also always used in cases of parasitic collateral feeders. Selective or superselective embolization was performed with the use of drug-eluting agents. Loading of the beads required 20-60 minutes and was done before starting the catheterization. DC-Beads (Biocompatibles, Surrey, United Kingdom) with a diameter of 100-300 μm were loaded with 25 or 37.5 mg of doxorubicin (Pfizer)/mL of suspension to a maximum of 150 mg of doxorubicin-loaded. Doxorubicin-eluting embolic agents were mixed with nonionic contrast material before intra-arterial administration. If needed to achieve intratumoural vessel occlusion, microspheres with a diameter of 100-300 μm and/or 300-500 μm (Embosphere; Merit Medical, South Jordan, UT, United States) or absorbable gelatin sponge particles (GelitaSpon; Gelita Medical BV, Amsterdam, The Netherlands) mixed with iodine contrast agent before intra-arterial administration were used.

The TACE endpoint was significant flow reduction while avoiding stasis in the artery feeding the tumour (ie, the contrast column was aimed to be cleared within 2-5 heartbeats). Selectivity of embolization was achieved in all patients. No procedure-related death within 2 months and no technical peri-procedural complications were observed (arterial dissections, bleeding, or infections).

### Endpoint and reference standard

The primary endpoint was the complete tumour response rate measured by modified Response Evaluation Criteria in Solid Tumours (mRECIST)^15^ on contrast-enhanced CT (reference standard) within 2 months following treatment. Patients were classified as complete responders if all treated lesions demonstrated CR as defined by mRECIST. Patients with partial response, stable disease, or progressive disease were pooled in opposition to complete responders. All cases were scored independently first by 2 subspecialized abdominal radiologists (JD and JG) blinded to the TACE procedure, and then reviewed together by the readers after the individual evaluation. Any disagreement between the readers was discussed until a final consensus was generated.

### Segmentation/annotation

Tumour segmentation was performed by 2 subspecialized abdominal radiologists, first by JD and then corrected in consensus by JG on pre-treatment 1.25 mm arterial phase and 1.25 mm portal venous phase CT images using 3D Slicer.^16^

### Deep learning model development

**Figure 2.**
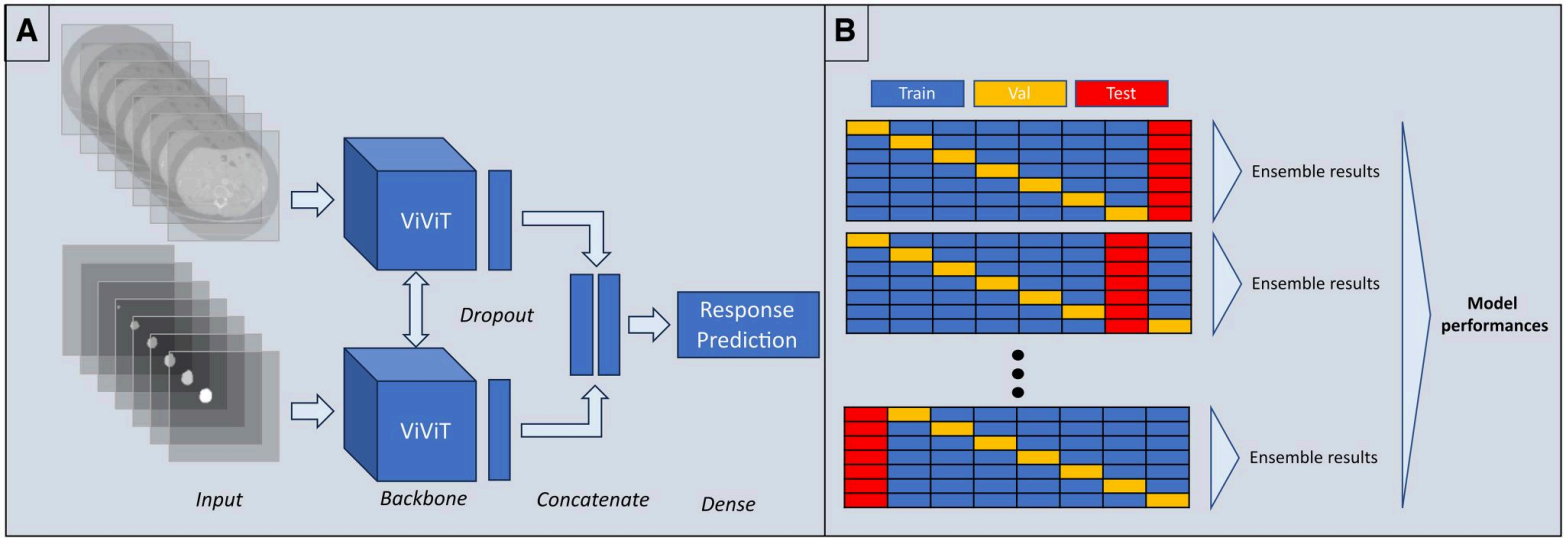
Twin deep learning model with spatio-temporal ViViT (Video Vision Transformer) as backbone and ensemble testing approach. (A) The model with 2 3D volumes as independent inputs. The first volume was composed of either late arterial or portal venous phase images, arranged sequentially into a 3D tensor. The second input volume was the corresponding 3D tumour segmentation. The model generated a predictive score ranging from 0 to 1, where a score of 1 corresponds to a complete response, while a score of 0 indicates any alternative outcome. (B) The extensive training/testing method based on an 8-fold cross-validation approach with an ensemble technique, a model vote system where the outcome is the average of multiple model predictions.

A twin DL model was employed to predict the probability of a CR to the treatment (Figure 2). This model utilized 2 3D volumes as independent inputs. The first volume comprised either late arterial or portal venous phase images arranged sequentially into a 3D tensor. The second input volume was the corresponding 3D tumour segmentation. The model generated a predictive score ranging from 0 to 1, where a score of 1 corresponds to a CR, while a score of 0 indicates any alternative outcome. A spatio-temporal ViViT, a transformer-based architecture designed for 3D input,^17^ served as a backbone for the model. ViViT was configured with 8 attention heads, 8 transformer layers, and an embedding dimension of 128. To mitigate potential memory constraints of the available hardware, a deliberate decision was made to reduce the size of the input volume to 128 × 128×128. For the same reason, the 2 ViViT heads were configured to share weights, as fewer parameters require less data to train. Consequently, the model included the twin ViViT heads, followed by a dropout layer (with a dropout rate of 0.5), concatenation of the embeddings from the 2 backbone heads and a fully connected layer of size 1 with Sigmoid activation, the output of which corresponds to the model’s prediction. During training, the input volumes were augmented with random rotation, elastic transform, and Gaussian noise to diversify the training set and enhance the algorithm’s generalization capability. To further prevent overfitting, label smoothing of 0.1 was applied to the optimizer, which was experimentally chosen to be Adam with a learning rate of 4.16e−04.

### Statistical analysis

An extensive training/testing method was required to validate the results to overcome the challenge of such a limited and imbalanced dataset. The model was evaluated using an 8-fold cross-validation approach with the ensemble method, a model vote system where the outcome is the average of multiple model predictions. Because the data was split into 8 folds, it was possible to use each split as a distinct test set, leveraging all available data for test coverage. Additionally, 7 separate models were trained for each test split, with the final prediction being the average of each of the 7 predictions. This scheme resulted in a thorough 56-model testing procedure where 8 ensemble models, each aggregating over 7 models, were averaged to obtain the model diagnostic performances (sensitivity, specificity, balanced accuracy, Area Under the Receiver Operating Characteristic—AUROC, average precision, and F1 score).

## Results

### Population

The final study population consisted of 93 patients (median age, 65 years; range 25-89 years) in both institutions: 44/93 patients (47%) with 50 lesions treated were included from institution 1; 49/93 patients (53%) with 49 lesions treated were included from institution 2. Six out of the 93 included patients had 2 distinct focal tumours treated by TACE during the same session. The CR rate was 36% (18/50) and 22% (11/49) at Institutions 1 and 2. Table 1 summarizes the clinical and biological profiles of the patients. Most patients at both institutions had Child-Pugh A or B cirrhosis, 93% (41/44) at Institution 1 and 76% (37/49) at Institution 2. Chronic hepatitis C was the leading cause of chronic liver disease (21/44-48%—at Institution 1 and 15/49-31%—at Institution 2), followed by Metabolic dysfunction Associated Steatotic Liver Disease (MASLD) and chronic hepatitis B. Median α-fetoprotein was 12 ng/mL at Institution 1 and 23 ng/mL at Institution 2.

**Table 1.** Main baseline demographic and clinical characteristics of patients in the training and validation cohorts.

	Institution 1 (*n *= 44)		Institution 2 (*n *= 49)	
Characteristic	Complete response	Incomplete response	Complete response	Incomplete response
	(*n *= 14)	(*n *= 30)	(*n *= 11)	(n = 38)
**Sex**				
Male	9 (64)	23 (77)	10 (91)	28 (74)
Female	5 (36)	7 (23)	1 (9)	10 (26)
**Median Age (years)**	67 [56-75]	65 [58-77]	63 [61-70]	65 [57-74]
**Cause of chronic liver disease**				
Alcohol	2 (14)	4 (13)	0 (0)	5 (13)
Chronic hepatitis B	4 (29)	4 (13)	2 (18)	5 (13)
Chronic hepatitis C	8 (57)	14 (47)	4 (36)	11 (29)
MASLD	4 (29)	7 (23)	2 (18)	5 (13)
MetALD	0	1 (3)	1 (9)	2 (5)
Unknown	0	0	0	2 (5)
No chronic liver disease	0	0	2 (18)	4 (11)
**Cirrhosis**	14 (100)	27 (90)	9 (82)	29 (76)
**Child-Pugh stage**				
A	14 (100)	20 (74)	7 (78)	24 (83)
B	0	7 (26)	2 (22)	5 (17)
**Biochemical analysis**				
Albuminemia (g/dL)	41 [32-43]	33 [32-38]	40 [36-41]	39 [32-42]
Prothrombin ratio (%)	102 [99-103]	100 [96-103]	80 [67-93]	82 [71-98]
Total bilirubinaemia (mg/dL)	14 [12-19]	22 [15-31]	12 [12-22]	15 [11-28]
α-fetoprotein (ng/mL)	19.5 [5.3-47.3]	19.0 [7.3-67.3]	3.0 [2.0-7.0]	14.0 [5.5-45.3]
**Hepatocellular carcinoma treated by TACE**				
Median number of HCC tumours per patient	1 [1-2]	1 [1-2]	1 [1-1]	1 [1-11]
Median size of HCC tumour (mm)	22 [17-35]	46 [37-53]	38 [27-54]	41 [36-44]
Number of HCC tumours	17	32	11	38
**CT scanners**				
GE Revolution EVO	8	11	0	0
GE LightSpeed VCT	4	18	11	38
GE Revolution HD	0	0	0	0
Philips Ingenuity	2	0	0	0
Philips Brilliance 64	0	1	0	0

Abbreviations: HCC = hepatocellular carcinoma; MASLD = metabolic dysfunction associated steatotic liver disease; MetALD = MASLD and alcohol-associated liver disease; TACE = transarterial chemoembolization.

Percentages are in parentheses and interquartile range in squared brackets.

### Tumour characteristics and treatment response

The median number of HCC tumours treated by TACE per patient was 1 (range 1-2) at both institutions with a total of 50 HCC tumours treated at Institution 1 and 49 at Institution 2. The median [range] tumour size was 40 mm [18-61] at Institution 1 and 37 mm [10-53] at Institution 2. The CR rate was 36% (18/50) at Institution 1 and 22% (11/49) at Institution 2.

### Model performance metrics

**Figure 3.**
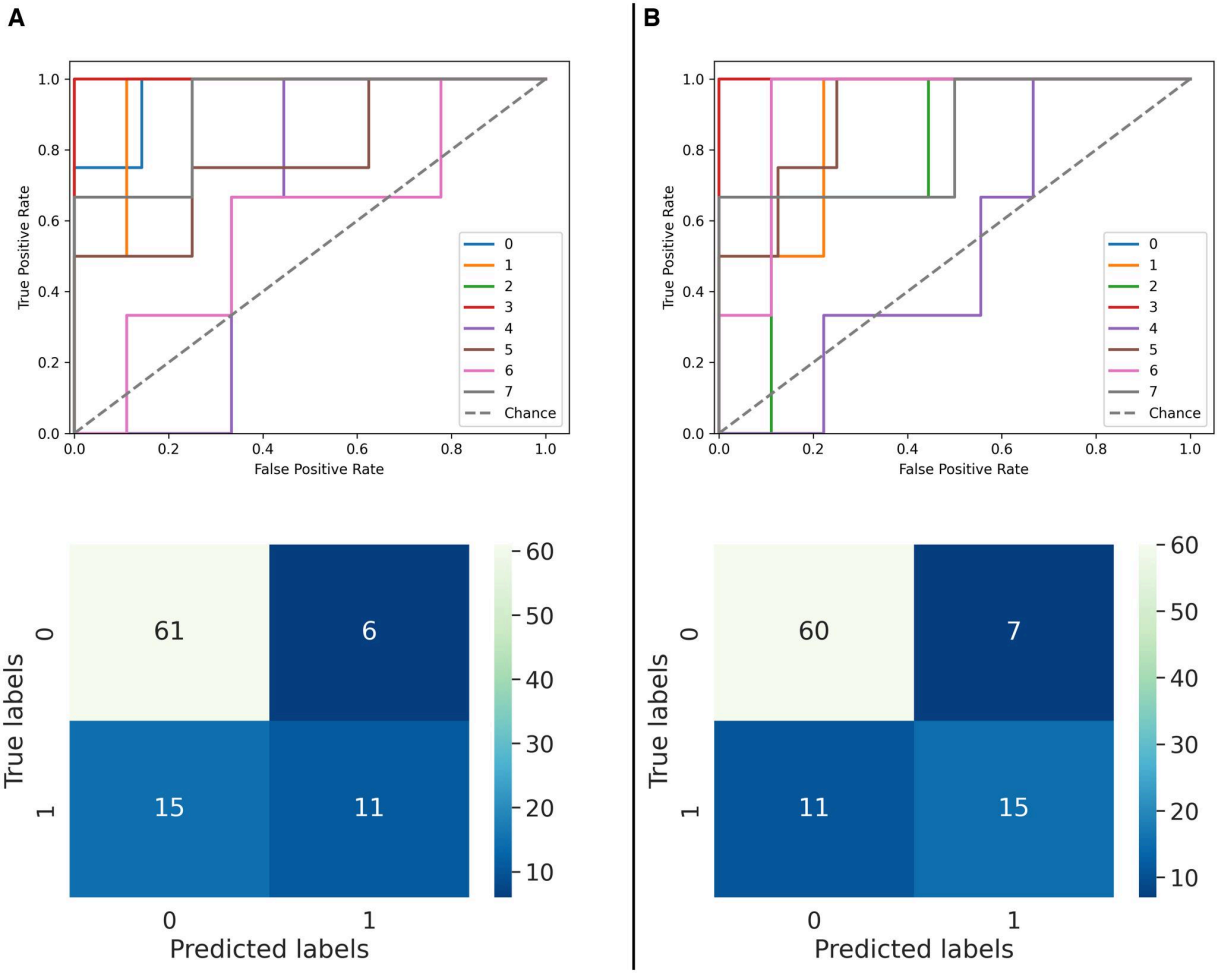
Area under the receiver operating characteristics curves and confusion matrices of the model with the late arterial phase (A) and the portal venous phase (B) as input.

**Figure 4.**
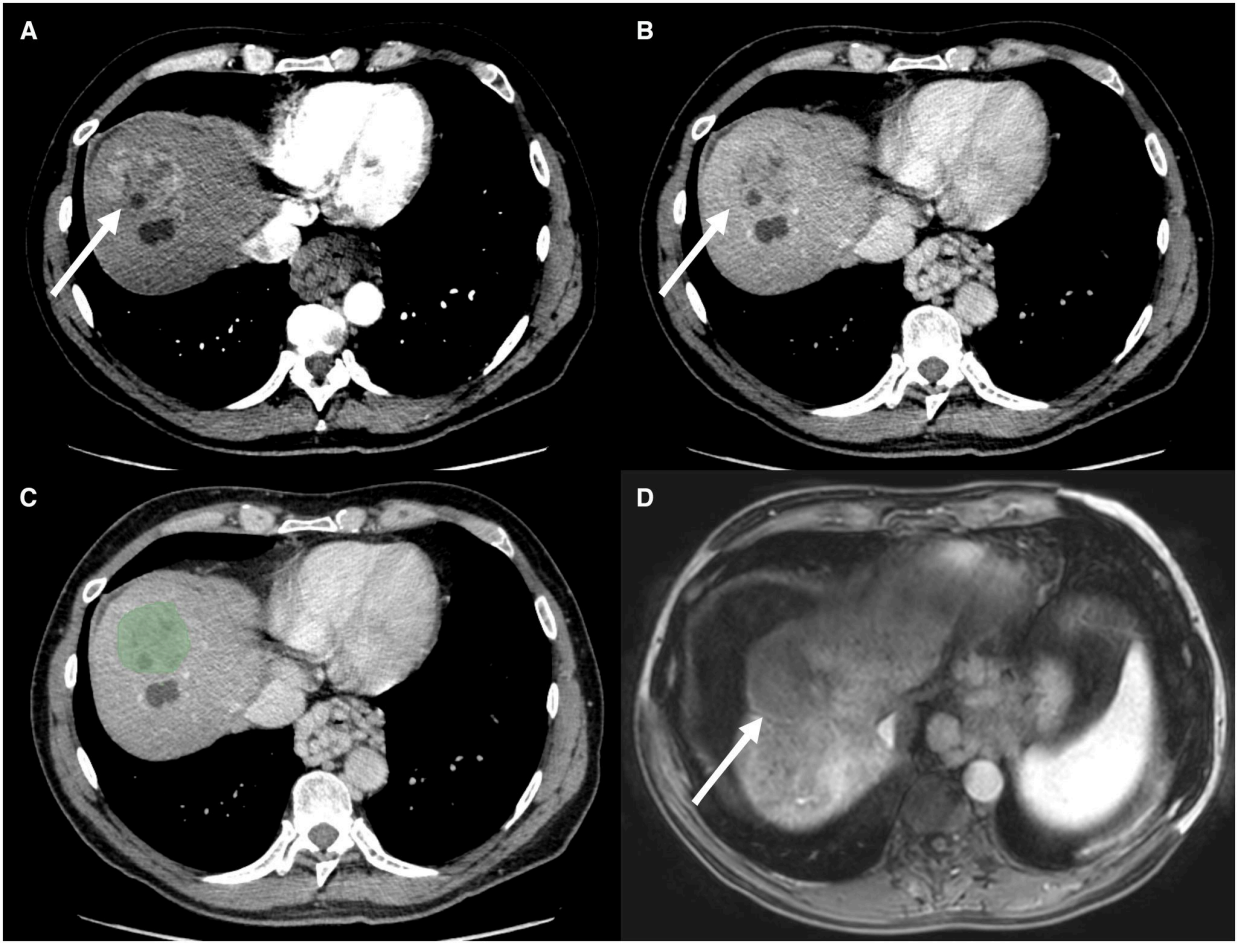
Complete response of a 53 mm hepatocellular carcinoma (arrow) in a 62-year-old male patient with a metabolic dysfunction associated steatotic liver disease (MASLD) cirrhosis (Child Pugh A5). (A) Illustrates a late arterial phase CT acquisition, (B) a portal venous phase CT acquisition, (C) the latter with superimposed tumoural segmentation, and (D) the post-treatment late arterial MRI phase. Complete response was correctly predicted by both models with a score of 0.64 for the late arterial model and 0.52 for the portal venous model.

**Figure 5.**
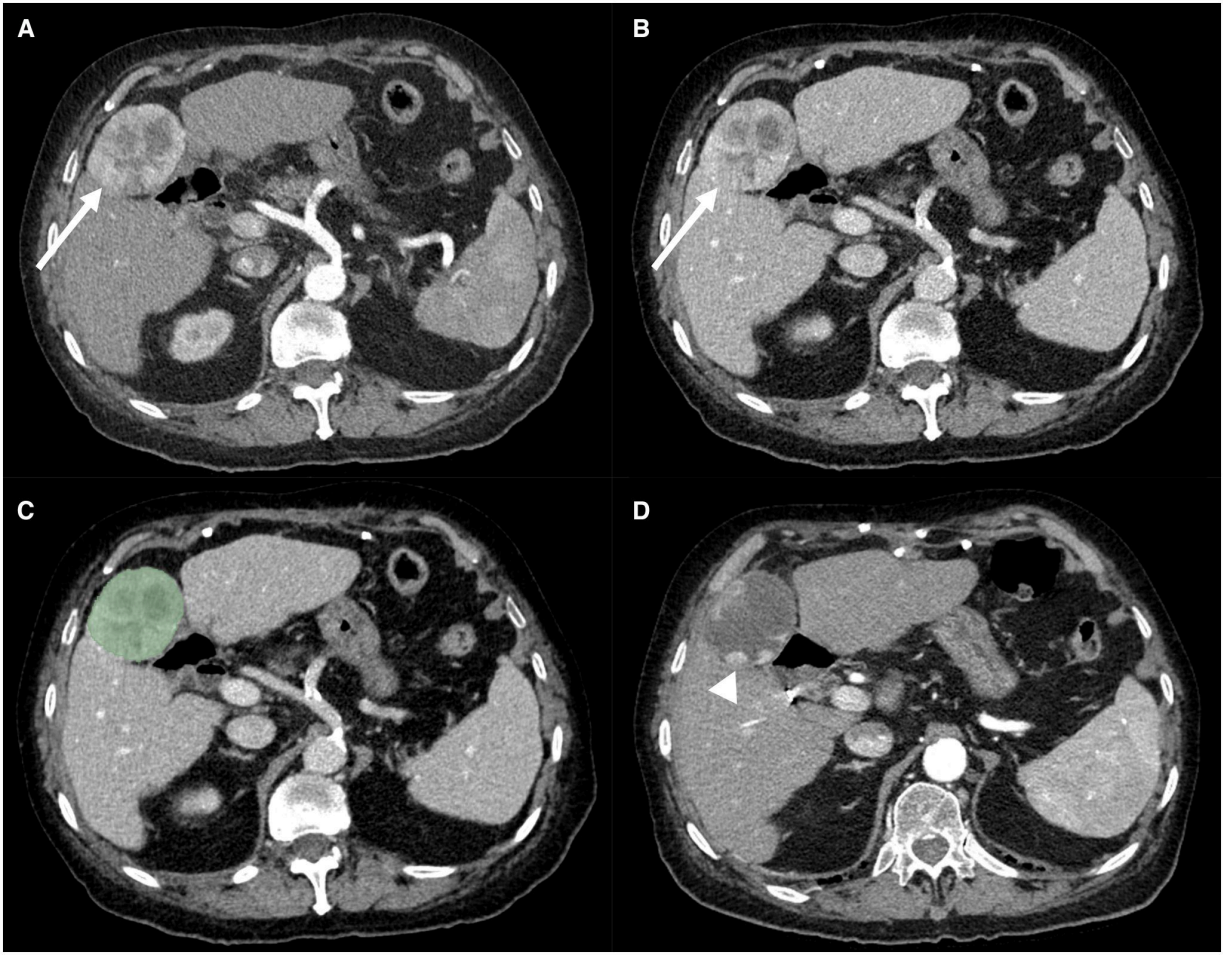
Partial response of a 60 mm hepatocellular carcinoma (arrow) in an 84-year-old male patient with hepatitis C virus cirrhosis (Child-Pugh A6). (A) Illustrates a late arterial phase CT acquisition, (B) a portal venous phase CT acquisition, (C) the latter with superimposed tumoural segmentation, and (D) the post-treatment late arterial phase at 2 months. The absence of complete response (residual tumour—arrowhead) was correctly predicted by both models with a score of 0.24 for the late arterial model and 0.17 for the portal venous model.

Table 2 summarizes the diagnostic performances of the model. The model achieved high specificity and AUC (Figure 3), good balanced accuracy, and moderate sensitivity when using either the late arterial phase (0.91 ± 0.12, 0.86 ± 0.16, 0.67 ± 0.13, 0.43 ± 0.23, respectively) or the portal venous phase (0.90 ± 0.15, 0.85 ± 0.17, 0.74 ± 0.16, 0.57 ± 0.30, respectively) with the manual tumoural segmentation. Figures 4 and 5 illustrate cases of CR and partial response.

**Table 2.** Model performances in the testing dataset using the ensemble method.

	F1 (%)	Average precision	AUROC	Balanced Accuracy	Specificity	Sensitivity	PPV	NPV
Late arterial phase	0.48 ± 0.26	0.76 ± 0.26	0.86 ± 0.16	0.67 ± 0.13	0.91 ± 0.12	0.43 ± 0.23	0.61 ± 0.38	0.81 ± 0.07
Portal venous phase	0.58 ± 0.32	0.77 ± 0.26	0.85 ± 0.17	0.74 ± 0.16	0.90 ± 0.15	0.57 ± 0.30	0.62 ± 0.36	0.85 ± 0.10

The model was evaluated using an 8-fold cross-validation approach with an ensemble method, a model vote system where the outcome is the average of multiple model predictions. Because the data was split into 8 folds, it was possible to use each split as a distinct test set, leveraging all available data for test coverage.

Abbreviations: AUROC = area under the receiver operating characteristic; NPV = negative predictive value; PPV = positive predictive value.

## Discussion

Over the past 2 decades, predicting the response to TACE in intermediate-stage HCC has remained a significant challenge. This study’s innovative use of DL techniques for predicting CR to DEB-TACE reflects the evolution of precision medicine. The model exhibited balanced accuracies of 67% and 74% on late arterial and portal venous phases, respectively, with a specificity of 91% and 90%, suggesting its potential utility in clinical settings. However, the model’s sensitivity of 43% and 57% indicates a need for refinement, particularly in accurately identifying all positive responders.

This research contributes to the growing integration of ML and DL in predicting TACE responses, as delineated in the comprehensive review by Hsieh et al.,^18^ which included 17 studies, mainly from China with chronic hepatitis B as the most common aetiology of chronic liver disease, published between 2018 and 2022, with a median cohort size of 194 patients. The geographic concentration of these studies sheds light on the potential regional variations in HCC aetiology and treatment response, emphasizing the need for diverse, multicentre datasets to ensure AI models’ generalizability in healthcare settings. The reliance on contrast-enhanced CT images in this study parallels the predominant imaging technique in the field, offering a benchmark for comparison. However, the comparison is somewhat limited, as most studies present a high risk of bias according to the Quality Assessment of Diagnostic Accuracy Studies (QUADAS)-AI score.^19^

In 2019, Morshid et al. reported a 74.2% [64%-82%] accuracy rate [95%CI] in predicting time to progression in a study focusing on mainly BCLC C patients.^12^ This differs from the present study’s endpoint of tumour CR rate, assessed by mRECIST on contrast-enhanced CT at 2 months following DEB-TACE. Although radiologically relevant, this endpoint can be subject to criticism as clinical trial outcomes are overall survival and progression-free survival. Yet, our goal was to assess the capacity to predict a CR and not to have outcomes affected by subsequent therapies.^20^ Early assessment of post-TACE tumour response, according to mRECIST, correlates with survival outcomes.^6^

The study’s alignment with the BCLC 2022 criteria underscores its relevance to current therapeutic strategies. Accurate prediction of treatment response could strengthen the position of TACE among the multiple currently available therapeutic options. This would result in better therapeutic strategies, as illustrated by recent studies on the interest of adjuvant immunotherapy.^21^ Despite its retrospective nature and modest dataset size, which the specific study’s inclusion criteria can explain, the inclusion of various imaging scanners and a multicentric approach enhances the robustness of the data, reflecting real-world clinical diversity while maintaining an adequate balance between HCC with or without CR. A key strength is the use of a state-of-the-art DL model, aligning with the latest advancements in AI model training. This study demonstrates an overall low risk of bias as per QUADAS-AI (Patient selection: Low; Index test: Unclear; Reference standard: Low; Flow and timing: Low). However, although it allowed extensive training and testing, the employed method of 8-fold cross-validation with an ensemble method, representing a model vote system where the outcome is the average of multiple model predictions, is a limitation to the model’s generalizability. Given the modest dataset size, merging both institutions’ cohorts and using this alternative approach to external independent testing were needed. In addition, larger datasets are needed to further improve the model performances. Although the model achieved high specificity allowing strong confidence in the response to DEB-TACE in the patients identified by the model, the moderate sensitivity would result in inadequate therapeutic management for a significant number of patients. Patients with BCLC B HCC that would completely respond to DEB-TACE would not be identified.

Furthermore, the evaluation methodology, though rigorous, cannot fully address the variability inherent in clinical practice, particularly considering the operator-dependent nature and challenging standardization of TACE interventions. This calls for cautious optimism regarding the current utility of the model and highlights the importance of incorporating procedural variability in future predictive modelling efforts. Future research should focus on integrating this variability, aiming for more robust AI applications in predicting TACE responses, thereby enhancing personalized treatment strategies in HCC.

## Conclusion

Our DL model, grounded in current clinical practice guidelines, represents a step towards refining DEB-TACE response prediction in HCC. Yet, it also highlights the difficult prediction of interventional oncology treatments. As these treatments are multistep and difficult to standardize, future research should aim not only to expand the training datasets but also to incorporate prospective studies to enhance the model’s sensitivity and predictive power.
